# Proteomic analysis of an *Aedes albopictus *cell line infected with Dengue serotypes 1 and 3 viruses

**DOI:** 10.1186/1756-3305-4-138

**Published:** 2011-07-18

**Authors:** Sirilaksana Patramool, Pornapat Surasombatpattana, Natthanej Luplertlop, Martial Sévéno, Valérie Choumet, Frédéric Thomas, Dorothée Missé

**Affiliations:** 1 Laboratoire Maladies Infectieuses et Vecteurs: Ecologie, Génétique, Evolution, Contrôle., UMR 5290 CNRS/IRD/UM1, Montpellier, France; 2 Department of Tropical Hygiene, Faculty of Tropical Medicine, Mahidol University, Bangkok, Thailand; 3 Plate-forme de Protéomique Fonctionnelle, IFR3, CNRS-UMR 5203, INSERM-U661, UMI-II, 34094 Montpellier France; 4 Unité de Génétique Moléculaire des Bunyavirus, Institut Pasteur, Paris, France

## Abstract

**Background:**

Proteomic analysis was performed to identify proteins regulated during infection by Dengue serotypes 1 and 3 in an *Aedes albopictus *cell line. The potential of these viruses to cause severe disease at primary infection is of interest although few studies have been performed with these two Dengue serotypes.

**Results:**

The most relevant observation of our study is the significant overexpression of proteins involved in the cellular stress response and the glycolysis pathway after 48 hours of infection. Viral infection activates the *translation *of some *host *genes, which may result in stress due to responses involving unfolded proteins.

**Conclusions:**

Therefore, the oxidation reduction and glycolytic mechanisms could participate in the antiviral response against Dengue virus. The results of our study should help to improve our knowledge of the virus-mosquito interaction at a cellular level with the aim of designing efficient strategies for the control of Dengue virus.

## Background

*Aedes aegypti *(Diptera, Culicidae) is considered the major vector for Dengue infection outbreaks worldwide [1]. *Aedes albopictus *(Diptera, Culicidae) is a less efficient vector for this virus, although it was involved in Dengue outbreaks in Japan, Seychelles, Hawaii, and Reunion Island [2]. The recent invasion of this second vector into America, Europe, and Africa could increase the transmission of arboviruses in tropical as well as temperate regions [3,4]. Dengue virus (DENV) can cause several clinical forms, ranging from an asymptomatic disease to severe Dengue hemorrhagic fever (DHF) or Dengue shock syndrome [5]. The World Health Organization estimates that 2.5 billion people live in more than 100 endemic areas where DENV can be transmitted [5]. DENV dramatically expands each year into new territories [6,7] as a consequence of combined factors such as the rapid and easy mobility of human populations, the distribution of mosquito vectors, and the lack of herd immunity in unexposed populations [8].

There are four serotypes of DENV (DENV-1 to DENV-4) that differ by their antigenic groups. Each group can be divided into three to five different genotypes. Therefore, it is difficult to take into consideration all these factors when trying to determine the mechanisms involved in the pathogenesis of the virus. Many researches have focused on DENV-2, which provokes the most severe form of Dengue in secondary infection [8,9], but types 1 and 3 have recently been found as new emerged types in Europe and Africa [2,10]. The report of two autochthonous DENV-1 infected persons in metropolitan France in September 2010 is a recent example of the introduction and local transmission of DENV outside its traditional area [2]. Many studies have reported that DENV-1 and DENV-3 cause severe disease at primary infection while DENV-2 and DENV-4 are frequently involved in Dengue outbreaks at secondary infection [11-14].

Because there is no available antiviral treatment or vaccine to cure or prevent DENV, other approaches are needed to fight and control the virus. A good understanding at the molecular level of the virus-mosquito interaction should help with the design of efficient strategies for the control of DENV. Mosquito cell infection is a part of the Dengue viruses' life cycle that is poorly understood. In this study, we compared the proteome of infected and non-infected cultures of the C6/36 *Ae. albopictus *cell line using two-dimensional differential in-gel electrophoresis (2D-DIGE) to examine modulated proteins of infected mosquito cells. We chose DENV-1 and DENV-3 as study models because of their remarkable capacities to induce severe disease with the first infection. Furthermore, travelers have more chance to develop DENV-1 or DENV-3 infection when they live in non-endemic areas and have never been in contact with the virus [2,10,15]. This could be due to the lack of herd immunity in this human population [16].

## Results

### 2-D-DIGE analysis of dengue infected and non-infected C6/36 Ae. albopictus cells

**Figure 1:**
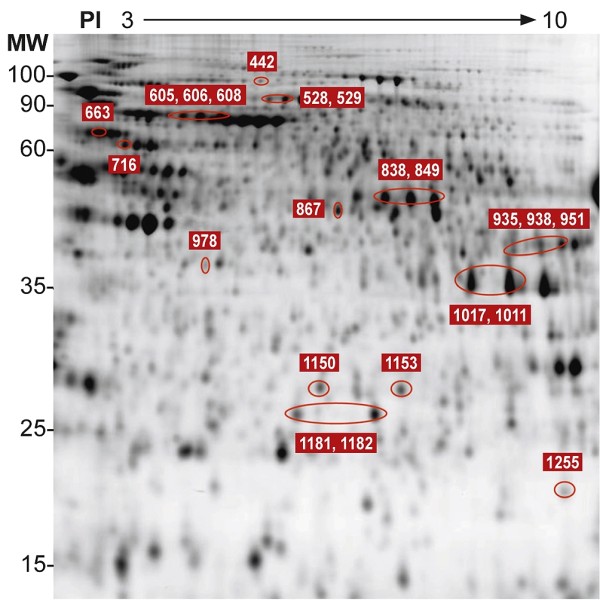
**2D-DIGE synthetic gel of *Ae. albopictus *C6/36 cells infected or not with DENV-1 and DENV-3**. Proteins were run with a pI scale of 3-10. Protein spots differentially expressed (numbered spots) were identified by MALDI-TOF MS. The molecular weight scales are indicated in the figure.

C6/36 cells were collected 48 h after infection with DENV-1, DENV-3, or a control (hereafter "mock infected"). We chose to use 48 h post infection because it was previously shown that this time corresponded to the exponential phase for both DENV-1 and DENV-3 productions in C6/36 cells [17]. We assumed that most cells are infected by the virus at this time. Proteins were prepared and labelled to run on 2D-DIGE. About 1500 spots were detected on 24 cm Immobiline DryStrip, pH 3-10 NL, followed by SDS-PAGE electrophoresis (Figure 1). Our analysis of gel images, quantification of protein expression, and statistical analysis revealed 22 differentially expressed protein spots. Notably, the mosquito cells infected by DENV-1 or DENV-3 showed similar patterns of protein expression (i.e same trend for the expression of proteome) compared to mock infected cells. The protein expression patterns can be divided into three groups according to the level of protein expression (Table 1). Group I includes 2 proteins that are up-regulated in cells infected by DENV-1 compared with their expression in DENV-3 infected and mock-infected cells (Table 1). The second group is composed of 15 proteins, the expression of which is up-regulated in DENV-3 infected cells compared to DENV-1 infected and mock-infected cells (Table 1). In these two groups, the protein expression is higher in DENV-infected than in the mock-infected cells (Table 1). The third group has 5 proteins that are up-regulated in mock-infected cells compared with the DENV-infected ones (Table 1).

**Table 1:** Differential protein expression between an uninfected and DENV-infected C6/36 *Ae.albopictus *cell line

Spot N°	ANOVA (p)	Fold	Average Normalized Volumes			Functional annotation
				
			DENV 1	DENV 3	CONTROL C6-36	
**Group I: DV1>DV3>C**						

978	8.98E-13	2.6	1.594	1.53	0.605	Putative uncharacterized protein

663	2.88E-09	2.6	1.604	1.183	0.613	Quinone oxidoreductase

**Group II: DV3>DV1>C**						

838	9.00E-11	4.3	1.114	1.79	0.421	Enolase

849	5.34^E ^-13	3.3	1.395	1.947	0.584	Enolase

606	3.16E-07	3.2	1.111	1.323	0.417	Putative uncharacterized protein

1153	6.09E-10	2.9	1.235	1.584	0.549	Phosphoglycerate mutase

608	3.21E-07	2.8	1.305	1.429	0.507	Putative uncharacterized protein

1181	1.50E-10	2.7	1.18	1.559	0.57	Triosephosphate isomerase

935	5.15E-10	2.7	1.251	1.569	0.579	Fructose-bisphosphate aldolase

1150	5.47E-11	2.5	1.237	1.551	0.608	Phosphoglycerate mutase

605	2.57E-06	2.3	0.898	1.094	0.483	Putative uncharacterized protein

716	3.46E-05	2.3	0.962	1.148	0.489	Chaperonin-60kD

1182	1.52E-13	2.2	1.32	1.541	0.703	Triosephosphate isomerase

938	1.39E-11	2.2	1.214	1.477	0.684	Fructose-bisphosphate aldolase

951	3.83E-11	2.2	1.113	1.454	0.67	Fructose-bisphosphate aldolase

1017	3.84E-10	2.1	1.002	1.367	0.653	Glyceraldehyde-3-phosphate dehydrogenase

1011	4.09E-08	2.1	0.951	1.313	0.612	Glyceraldehyde-3-phosphate dehydrogenase

**Group III: C>DV1 and DV3**						

1255	1.65E-12	2.0	0.814	0.758	1.509	Calponin

529	1.51E-10	3.1	0.49	0.605	1.511	Procollagen-lysine,2-oxoglutarate 5-dioxygenase

528	2.32E-13	2.8	0.476	0.618	1.326	Procollagen-lysine,2-oxoglutarate 5-dioxygenase

867	2.69E-10	2.0	0.672	0.74	1.328	Ethanolamine-phosphate cytidylyltransferase

442	9.77E-08	2.0	0.763	0.834	1.500	Aconitase

### Identification of candidate proteins

Twenty two spots were identified as being 13 different proteins by Matrix assisted laser desorption ionisation time-of-flight mass spectrometry (MALDI-TOF MS) (Table 2). Several of these spots were identified as the same protein. These different forms of a protein could be due to post-translational modifications like glycosylation, phosphorylation, acetylation, or protein degradation.

**Table 2:** Protein identification by MALDI-TOF MS

N° spot DIGE	Entree Swissprot_TrEMBL	Identification	Biological process	Cellular component	Molecular function	Sequence similarities	Pathway	Molecular mass (Da)	p*I*	MASCOT score	Cover sequence
Protein up-regulated in infected cells											

978*	Q17AU4_AEDAE	Putative uncharacterized protein						42429	6.5	82	25

663*	Q16S95_AEDAE	Quinone oxidoreductase	Oxidation reduction		Oxidoreductase activity, zinc ion binding			50810	5.6	121	31

838°, 849°	Q17KK5_AEDAE	Enolase	Glycolysis	Cell surface, phosphopyru-vate hydratase complex	Magnesium ion binding, phosphopyruvate hydratase activity	Belongs to the enolase family.	Carbohydrate degradation, glycolysis, pyruvate from D-glyceraldehyde 3-phosphate: step 4/5	46877	6.3	202	53

605°, 606°, 608°	B0WBF6_CULQU	Putative uncharacterized protein	Protein folding		Chaperone, ATP binding, unfolded protein binding	Belongs to the heat shock protein 70 family		72845	5.9	188	32

1150°, 1153°	Q177P3_AEDAE	Phosphoglycerate mutase	Glycolysis		2,3-bisphosphoglycerate-dependent phosphoglycerate mutase activity			28582	6.6	131	41

1181°, 1182°	Q17HW3_AEDAE	Triosephosphate isomerase	Glycolysis		Isomerase, triose-phosphate isomerase activity	Belongs to the triosephosphate isomerase family	Carbohydrate biosynthesis, gluconeogenesis, carbohydrate degradation, glycolysis, D-glyceraldehyde 3-phosphate from glycerone phosphate: step 1/1	26705	6	161	44

935°, 938°, 951°	Q178U8_AEDAE	Fructose-bisphosphate aldolase	Glycolysis		Fructose-bisphosphate aldolase activity	Belongs to the class I fructose-bisphosphate aldolase family.	Carbohydrate degradation, glycolysis, D-glyceraldehyde 3-phosphate and glycerone phosphate from D-glucose: step 4/4	39551	8	172	50

716°	Q16PM9_AEDAE	Chaperonin-60kD	Protein refolding	Cytoplasm	ATP binding, unfolded protein binding, chaperone	Belongs to the chaperonin (Hsp60) family		61155	5.5	75	20

1011°, 1017°	B0WEB5_CULQU	Glyceraldehyde-3-phosphate dehydrogenase	Glycolysis, oxidation reduction	Cytoplasm	NAD or NADH binding, glyceraldehyde-3-phosphate dehydrogenase (phosphorylating) activity	Belongs to the glyceraldehyde-3-phosphate dehydrogenase family	Carbohydrate degradation, glycolysis, pyruvate from D-glyceraldehyde 3-phosphate: step 1/5	35693	8.5	114	40

Protein up-regulated in non-infected cells											

1255^#^	Q1HR19_AEDAE	Calponin						20936	8.3	183	73

528^#^, 529^#^	Q0IER9_AEDAE	Procollagen-lysine,2-oxoglutarate 5-dioxygenase	Oxidation reduction		L-ascorbic acid binding, iron ion binding, oxidoreductase activity acting on paired donors with incorporation or reduction of molecular oxygen, oxidoreductase activity acting on single donors with incorporation of molecular oxygen, incorporation of two atoms of oxygen			82324	5.7	202	25

867^#^	Q179F9_AEDAE	Ethanolamine-phosphate cytidylyltransferase	Biosynthetic process		Nucleotidyltransferase activity			42278	6.1	102	23

442^#^	Q16ZG5_AEDAE	Aconitase	Metabolic process		4 iron, 4 sulfur cluster binding			99252	5.8	165	23

Symbol : * represents proteins that are up-regulated in cells infected by DENV-1, **° **represents proteins that are up-regulated in cells infected by DENV-3 and **^# ^**represents proteins that are up-regulated in mock-infected cells.

Nine of the 13 identified proteins belong to the groups 1 and 2, i.e., those proteins that are up-regulated in infected conditions. Notably, five of these were enzymes involved in the glycolysis pathway. These included enolase, phosphoglycerate mutase, triosephosphate isomerase, fructose-bisphosphate aldolase, and glyceraldehyde-3-phosphate dehydrogenase (GAPD). The significant up-regulation of these enzymes in virus-infected cells might indicate that the virus disturbs glucose metabolism in host cells [18]. The four remaining overexpressed proteins were quinine oxidoreductase, chaperonin-60kD, and two putative uncharacterized proteins (Q17AU4_AEDAE and BOWBF6_CULQU). Quinone oxidoreductase is implicated in oxidation-reduction reactions; chaperonin-60kD and Q17AU4_AEDAE are involved in protein folding process; and BOWBF6_CULQU has no known function.

The four overexpressed proteins in mock-infected cells were procollagen-lysine 2-oxyglutarate 5-dioxygenase (PLOD), calponin, ethanolamine-phosphate cytidylyltransferase, and aconitase. PLOD is involved in the oxidation-reduction necessary for the biosynthesis of collagen [19]; calponin inhibits the ATPase activity of smooth muscle myosin [20]; ethanolamine-phosphate cytidylyltransferase participates in biosynthesis specifically by transferring phosphorus-containing nucleotide groups [21]; and aconitase is a tricarboxylic acid cycle enzyme that has a role in the regulation of iron metabolism [22].

## Discussion

DENV usually induces apoptosis in infected mammalian cells but causes less damage in mosquito cells [23]. The mechanisms involved in the interaction between DENV and insect cells have been described in recent studies [9,24-27], but these only concern the DENV-2 virus serotype. DENV-1 and DENV-3 have been less studied than DENV-2 and DENV-4. DENV-1 and DENV-3 have been found to cause DHF at first infection [12]. This finding as well as the recent report of DENV-1 introduction in France [2] and the re-emergence of DENV-3 in Sudan [28] attract our interest in these two virus serotypes. In this study, we compared the proteomes of C6/36 *Ae. albopictus *cells infected and non-infected with DENV-1 and DENV-3.

Cells infected with DENV-1 and DENV-3 exhibited many proteins that were differentially expressed compared to the mock-infected cells. These data may reveal cellular stress due to viral infection. In response to this stress, mosquito cells utilize antioxidant reactions combined with increased energy production to cope with the virus. The up-regulation of chaperone proteins in infected cells might be due to stress in the endoplasmic reticulum (ER) induced by viral infection; ER is where proteins are synthesized. Only proteins that are correctly folded by ER chaperones are transported to the Golgi apparatus. Misfolded and unfolded proteins lead to ER stress. Eukaryotic cells up-regulate ER chaperones and establish the cytoprotective mechanism known as the unfolded protein response (UPR) to degrade the unfolded protein [29]. Molecular chaperones are involved in preventing both newly synthesized polypeptide chains and assembled subunits from aggregating into nonfunctional structures [30]. Chaperone proteins were shown to interfere with virus assembly and replication in *Ae. albopictus *cells infected with Mayaro virus, and thus protect the cells from injury during viral infection [31]. Furthermore, the expression of Hsp90 was required for the activity of hepatitis B virus reverse transcriptase [32].

Interestingly, it has been reported that acute infection of mammalian cells with several types of viruses often results in the induction of heat-shock protein expression [33]. The up-regulation of chaperone proteins 24 hours after infection with DENV was previously reported [23]. These studies support our findings with infected cells, which show two up-regulated proteins involved in protein folding biological process: chaperonin-60kD (Q16PM9_AEDAE) and one putative uncharacterized protein identified by mass spectrometry as being from *Culex quinequefasciatus *(B0WBF6_CULQU). These proteins belong to the heat-shock protein families Hsp60 and Hsp70, respectively.

Cells require a chaperone function to prevent or correct misfolded or unfolded proteins created by environmental stress. These chaperone proteins aid protein folding in different ways. Hsp60 is a mitochondrial chaperone that generally assists the carrying and refolding of proteins from the cytosol to mitochondria [34]. Many Hsp70 chaperones could surround an unfolded substrate to stabilize it and prevent aggregation until the unfolded molecule folds properly, after which the Hsp70 chaperones will lose affinity for the molecule and diffuse away [34,35]. Hsp60 and Hsp70 could induce mitochondrial UPR in the same way as the ER stress response [29].

The modulation of chaperone-associated proteins could protect cells from apoptosis, as does the overexpression of calreticulin, which is a Ca^2+^-binding chaperone protein [36]. These findings suggest that the activation of these two chaperone proteins in infected cells due to oxidative stress could induce UPR to cope with ER or mitochondrial stress [23,29]. In turn, the increase of chaperone proteins may also be due to the subversion of the cell by the virus in order to complete viral replication. Indeed, it has been demonstrated that flavivirus infection activates the key transcription factor of the UPR and take advantage of this cellular response to alleviate virus-induced cytotoxicity [37].

Chen et al. [23] further demonstrated that mosquito cells use antioxidant mechanisms to survive DENV infection. The changes in mitochondrial membrane potential and the generation of superoxide confirm that DENV induces oxidative stress in C6/36 cells [23]. This supports our results showing the overexpression in infected cells of quinone oxidoreductase, which is increasingly recognized as the major contributor to reactive oxygen species formation [38]. NADH quinone oxidoreductase catalyses NADH to NAD^+^, reduces ubiquinone, and transports protons across the inner mitochondrial membrane. This enzyme complex also reduces O_2 _to superoxide, which causes cellular oxidative stress [38]. This means that mosquito cells use the oxido-reduction mechanism to protect themselves against DENV viral infection.

Another interesting result of our study is that five enzymes involved in the glycolysis pathway were up-regulated. Increased glucose uptake and glycolytic enzyme activity due to viral infection have been found in other studies [39,40]. The induction of glycolytic proteins involved in energy production is also found in CHIKV and DENV-2 infections [9]. Ritter et al. [18] suggested that the increased activity of glycolysis was due to the breakdown of the mitochondrial membrane, which decreased ATP concentration. As a result, the glycolysis pathway was activated to compensate for the lack of energy [18]. Recent studies have demonstrated more multifaceted functions of glycolytic enzymes such as GAPD and enolase. Both of these acquired non-glycolytic functions in transcriptional regulation. Moreover, GAPD might play a role as regulator or indicator of apoptosis [41]. Glycolysis is also reported to be the main source of energy production in *Trypanosoma*; this finding could represent a new drug target against parasites [42,43]. In addition, it has been reported that GAPDH binds to the minus-strand RNAs of Japanese encephalitis virus and the subcellular localization of GAPDH changed upon JEV infection, suggesting that GAPDH may play a role during the virus life cycle [44]. These evidences suggest that blocking the glycolysis pathway could constitute a new tool in the fight against mosquito-borne diseases *via *their vector control. The increase in glycolytic enzymes could also be a result of the arrest at the G1 cell cycle due to UPR.

## Conclusions

The modulation of protein expression found in our study might be the strategy of the virus to overcome host pathways to facilitate survival at the expense of the host. Further studies are needed to understand the mechanism by which these proteins are induced during viral infection. Virus could stimulate the transcription and translation of some host products for their survival, but the mechanism is still unknown. The modulation of protein expression could also be the mosquito's response to the viral infection. It would be interesting in a further study, to determine whether a similar response occurs in cells infected with another virus or if the observed modulation of protein expression is specifically induce in response to DENV infection. Mosquitoes generate oxido-reduction stress to cope with the virus and stimulate the glycolysis pathway to prevent cellular damage. Thus, it might be beneficial to understand the proteome of infected cells to develop an anti-pathogen approach. However, these proteomics studies need to be complemented by studies using RNAi gene silencing to allow the characterization of modulated genes *in vivo *in mosquito tissues. Improvements in our knowledge on mosquito cell systems will be important to decipher the infection process of dengue virus in human.

## Methods

### Cell culture and virus infection

*Ae. Albopictus *C6/36 cells were grown in minimal essential medium supplemented with 10% fetal calf serum, 1% L-glutamine, 1% sodium bicarbonate, 1% nonessential amino acids, 50 U/mL of penicillin, and 50 μg/mL of streptomycin at 28°C [45]. Cells were infected with two serotypes of Dengue virus, DENV-1 (Hawaii strain) and DENV-3 (H87 strain), at an multiplicity of infection of 0.01 then incubated at 28°C for 48 hours [17]. Mock-infected cells were used as the normal physiological control and represent cells incubated with supernatant of uninfected C6/36. The experiment was done in quadruplicate. The DENV-1 and DENV-3 virus stocks were propagated in C6/36 *Ae. albopictus *cells.

### Protein sample preparation

Infected or mock-infected cells were washed with PBS and then lysed by solubilizing buffer (7 M urea, 2 M thiourea, 4% CHAPS, 0.5% Triton X-100, and 40 mM Tris-HCl). After a centrifugation at 16000 × g for 45 min, the supernatants were collected and protein concentrations were measured using a 2-D Quant kit (GE Healthcare).

### 2D-DIGE, image scanning, and statistical analysis

For analytical 2D-DIGE, DENV-1 and DENV-3 infected and non-infected protein samples were compared using the CyDye DIGE Fluors for Ettan DIGE (Cy2, Cy3, and Cy5). Proteins were labelled according to the Ettan DIGE minimal labelling protocol (Ettan DIGE User Manual, GE Healthcare). For each sample (DENV-1, DENV-3 or mock-infected protein samples), 50 μg of protein was labeled with 400 pmol of either Cy3 or Cy5. The internal standard was a pool of equal amounts (25 μg) from all samples which was labeled with Cy2. These labeled samples were then combined and loaded on gels. Every gels contained 50 μg of sample labeled with Cy3, 50 μg of sample labeled with Cy5 and 50 μg of internal standard labeled with Cy2. The sample volume was made up to 450 μL by adding rehydration buffer (7 M urea, 2 M thiourea, 4% CHAPS, 0.5% Triton X-100, 40 mM Tris-HCl, 1% IPG buffer, and 1.2% DeStreak) prior to separation by isoelectric focusing (IEF). IEF was performed with 24 cm Immobiline DryStrip, pH 3-10 NL. The run conditions were as follows: rehydration for 14 h at 20°C, current of 50 μA per strip, 60 V (step) for 3 h, 1000 V (gradient) for 4 h, 8000 V (gradient) for 4 h, and 8000 V (step) until reaching a total of 64 000 Vh. 2-DE was performed on 12% SDS-PAGE gel at 15 mA/gel for 6 h and then at 30 mA/gel until the bromophenol blue front reached the end of the gel. Gels were scanned using a Typhoon 9400 imager (Amersham Biosciences). All gel images were acquired at 100 μm pixel resolution under nonsaturating conditions. 2D-DIGE images were analyzed using Progenesis SameSpots 3.1 software. Statistical analysis and protein quantification were carried out using this software with ANOVA which took into account the mean difference and the variance among 3 groups: DENV-1, DENV-3 and mock-infected groups. The fold change with a cut-off of 2.0-fold up- or down-regulated was used (calculated between the lowest mean normalised volume and the highest mean normalised volume of each spot). The statistical power of this study was greater than 0.8. Protein spots with a significant altered expression (p < 0.001) were trypsin digested and identified with mass spectrometry.

### Protein identification by MALDI-TOF MS

For 2D-DIGE, gels were run with 150 μg of a mix of protein from the different samples (50 μg of each sample labeled with Cy3, Cy5 and Cy2) and these gels were afterwards stained with CBB. Spots of interest were localized on the gels by comparing the CBB-stained spot pattern with the 2D-DIGE protein pattern. To ensure consistency, gels were analysed using Progenesis SameSpots 3.1 software and differential spots were identified. Gel image containing differential spots and picking gels were compared physically side-by-side and the candidate spots were excised manually in a laminar flow hood. Enzymatic in-gel digestion through peptide spotting and protein identification was performed as previously described [46,47]. Briefly, protein spots were digested using 150 ng of trypsin, peptide extraction was performed using five sonication cycles of 2 min each, and peptides were concentrated 1 h at 50°C; 0.5 μL of sample peptide and 0.5 μL of CHCA were deposited on a 384-well MALDI anchorship target using the dry-droplet procedure. Peptide samples were then desalted on the target using a 10 mM phosphate buffer. Analyses were performed using an UltraFlex I MALDI TOF-TOF mass spectrometer (Bruker Daltonics, Bremen, Germany) in the reflectron mode with a 26 kV accelerating voltage and a 50 ns delayed extraction. Mass spectra were acquired manually or in the automatic mode using the AutoXecute module of Flexcontrol (Bruker Daltonics) (laser power ranged from 30 to 50%, 600 shots). Spectra were analyzed using FlexAnalysis software (Bruker Daltonics) and calibrated internally with the autoproteolysis peptides of trypsin (m/z 842.51, 1045.56, 2211.10). Peptides were selected in the mass range of 900-3000 Da. Peptide Mass Fingerprint identification of proteins was performed by searching against the Insecta entries of either the Swiss-Prot or TrEMBL databases http://www.expasy.ch and by using the MASCOT v 2.2 algorithm (http://www.matrixscience.com) with trypsin enzyme specificity and one trypsin missed cleavage allowed [48]. Carbamidomethyl was set as fixed cystein modification and oxidation was set as variable methionine modification for searches. A mass tolerance of 50 ppm was allowed for identification. Matching peptides with one missed cleavage were considered as pertinent when there were two consecutive basic residues or when arginine and lysine residues were in an acidic context. MASCOT scores higher than 65 were considered as significant (p < 0.05) for Swiss-Prot and TrEMBL database interrogations.

## List of abbreviations used

Dengue Virus: DENV; *Aedes: Ae*; Japanese encephalitis virus: JEV; DHF: Dengue Hemorrhagic Fever; GAPDH: glyceraldehyde-3-phosphate dehydrogenase; UPR: Unfolded protein response; PLOD: Procollagen-lysine 2-oxyglutarate 5-dioxygenase; HSP: Heat shock protein; MALDI-TOF MS: Matrix assisted laser desorption ionisation time-of-flight mass spectrometry.

## Competing interests

The authors declare that they have no competing interests.

## Authors' contributions

Conceived and designed the experiments: SP, DM. Performed the experiments: SP, PS, MS. Analyzed the data: SP, PS, DM. Wrote the paper: SP, DM. Corrected the manuscript: VC, FT. All authors read approved the final version of the MS
